# Neural Markers Associated with the Temporal Deployment of Attention: A Systematic Review of Non-motor Psychophysical Measures Post-stroke

**DOI:** 10.3389/fnhum.2017.00031

**Published:** 2017-02-10

**Authors:** Essie Low, Robin Laycock, Sheila Crewther

**Affiliations:** Department of Psychology and Counselling, La Trobe UniversityMelbourne, VIC, Australia

**Keywords:** temporal attention, attentional deployment, neglect, stroke, psychophysical tasks

## Abstract

In recent years, evidence has emerged to suggest abnormal temporal dynamics of attentional processing in stroke patients, especially those presenting with neglect symptoms. However, there has been little profiling of the nature and extent of such temporal anomalies. In addition, many paradigms currently used to measure the time required to deploy visual attention in stroke require a psychomotor response, and may therefore confound performance outcomes. Thus, the aim of this systematic review was to identify and evaluate studies that have employed non-motor psychophysical paradigms to characterize the temporal deployment of visual attention in space. A total of 13 non-motor psychophysical studies were identified, in which stimulus exposure times were manipulated to measure the time course of attentional deployment. Findings suggest that prolonged attentional deployment thresholds are more likely to occur with lesions within more ventral areas of the fronto-parietal network, irrespective of whether patients presented with neglect. Furthermore, this deficit was greater following right-hemispheric lesions, suggesting a dominant role for the right-hemisphere in facilitating efficient deployment of attention. These findings indicate that area and hemisphere of lesion may serve as putative markers of attentional deployment efficiency. In addition, findings also provide support for using non-motor psychophysical paradigms as a more rigorous approach to measuring and understanding the temporal dynamics of attention.

## Introduction

The concept of attention has been broadly understood as being the behavioral process of concentrating on a particular task or information at hand, while filtering other ongoing activities within the perceptual environment (James, 1890). From electrophysiological and neuroimaging studies, attention has been conceptualized by distinct bottom-up (or exogenous) and top-down (or endogenous) processes (Correa et al., 2004; Peelen et al., 2004; Buschman and Miller, 2007; Shomstein et al., 2010; Cloutman, 2013; Joyce and Hrin, 2015). Specifically, exogenous attention has been defined as the allocation of attention that is driven by salient external sensory stimuli, and is neuroanatomically underpinned by more ventrally located fronto-parietal networks known as the Ventral Attention Network (Corbetta and Shulman, 2002; Shomstein, 2012). Endogenous attention, on the other hand, has been referred to as the allocation of attention that is driven by an individual's expectations, goals and knowledge, and is underpinned by more dorsal fronto-parietal tracts (i.e., Dorsal Attention Network; Corbetta and Shulman, 2002; Shomstein, 2012). In the case of unilateral spatial neglect, a common disorder of attention post-stroke, this phenomenon is known to be associated with an exogenous deficit in attending to salient stimuli within contralesional space, thus resulting in a lack of awareness to this side of visual hemi-space (Samuelsson et al., 1997; Mort et al., 2003; He et al., 2007; Bartolomeo et al., 2012). Interestingly, it is worth noting that the common neural regions that are implicated in neglect and its associated exogenous deficit (i.e., right temporo-parietal junction, right inferior parietal lobule, and inferior prefrontal gyrus) are also the same regions associated with the Ventral Attention Network (Husain and Kennard, 1996; Vallar, 2001; Mort et al., 2003; Umarova et al., 2011).

Attention can also be thought of in terms of its spatial and temporal properties, i.e., the notion that attention can occur in x, y, z coordinates of space, and over a time dimension. In this context, neglect has traditionally been associated with deficits of spatial attention—that is the inability to orient attention to a particular location in space (Friedrich et al., 1998; Karnath et al., 2002). Over the past decade, however, research has begun to demonstrate that neglect is not limited to spatial deficits alone. In fact, various forms of non-spatial, temporal deficits of attention have been demonstrated post-stroke, an example being the inability to orient attention to time properties, thus resulting in perceptual timing inaccuracies (e.g., underestimation of the passage of time; Harrington et al., 1998; Morin et al., 2005; Danckert et al., 2007; Merrifield et al., 2010; Calabria et al., 2011; Low et al., 2016). Overall, these studies have consistently suggested a role for a hypothetical internal clock (Treisman, 1963; Meck and Benson, 2002) that becomes compromised following a stroke, causing an inability to register the passage of time pulses as fast as the passage of real-time itself.

Central to the current review is another common form of temporal attentional deficit that has been demonstrated in neglect patients, known as temporal deployment of attention (Husain et al., 1997; Shapiro et al., 2002; Hillstrom et al., 2004). In contrast to the orientation of attention to time, temporal deployment of attention refers to the efficiency in deploying attention over time, when one attempts to attend to a particular stimulus in space (Husain et al., 1997; Coull, 2004). From the literature, this construct has also been referred to as the orientation of attention in space, but over the course of time. Importantly, characterization of the temporal deployment of attention may prove to be particularly useful since it provides a measure of how fast or efficient attention can be activated, deployed, and allocated to a particular object in space. From the literature, previous studies have predominantly employed computerized paradigms in neglect patients to explore this temporal deployment deficit (D'erme et al., 1992; Bartolomeo, 1997; Rorden et al., 1997; Snyder and Chatterjee, 2004; Ptak and Golay, 2006; Hamilton et al., 2010). Subsequently, findings generally revealed impaired performance in the form of prolonged reaction times to detect contralesional targets and impaired judgment of stimulus presentation order, indicating slower deployment (Rorden et al., 1997; Snyder and Chatterjee, 2004; Ptak and Golay, 2006). However, while these deployment deficits have been demonstrated, it is worthwhile noting that there remains a heterogeneity in the paradigms used (which raises the question of the validity of the construct being measured), as well as limitations that accompany particular paradigms themselves.

With regards to paradigm limitations, one example is the high frequency with which task methodology was adapted from the classic cued Posner paradigm, where the aim of the task was to investigate both spatial (i.e., attentional orienting) and temporal dynamics of attention (Rafal and Posner, 1987; D'erme et al., 1992; Friedrich et al., 1998; Rengachary et al., 2011; Wansard et al., 2015). Specifically, pre-target cues are deployed at varying onset asynchronies to investigate the timing of attentional deployment from one spatial location to another, thus limiting understanding of the time course of attentional deployment to a *particular* spatial location alone. Secondly, many currently used paradigms, including Posner's and other psychophysically-grounded tasks, require a psychomotor component to respond to task items (e.g., pressing the spacebar, clicking a mouse, saccadic/eye movement latency; D'erme et al., 1992; Erez et al., 2009; Ptak and Schnider, 2010; Rengachary et al., 2011; Cumming et al., 2012; Wansard et al., 2015). As a result, the outcome measure is likely to be significantly confounded by psychomotor reaction times especially when used with clinical groups that present with upper limb motor difficulties (as with stroke).

We would like to argue that an effective method to specifically quantify the time course of visually-driven deployment and allocation of attention without being confounded by psychomotor responses, is to utilize psychophysical methodologies that involve manipulation of a *time* variable pertaining to the presentation of the stimulus (Leek, 2001). This manipulation of time can take place in various forms, including, but not limited to: (1) varying the exposure duration of a stimulus to determine the minimal exposure time, or the threshold required for an individual to deploy and allocate attention for subsequent stimulus detection; or (2) varying the duration between presentations of two stimuli (i.e., stimulus onset asynchrony) to determine the duration interval required to most effectively detect the presence of the second stimulus or the order of stimuli presentation (Pelli and Farrell, 1995; Leek, 2001). As a result, both adaptive and non-adaptive psychophysics methodologies can be used to assess threshold or other aspects of the psychometric function relating to temporal processing unhindered by motor reaction time. With adaptive methods utilized for tasks such as the Inspection Time (Sadler and Deary, 1996) or Change Detection (Rensink, 2002; Rutkowski et al., 2003), the duration of stimulus presentation or duration interval is adjusted in accordance with participants' responses, allowing a threshold duration measurement. Alternatively, non-adaptive methods can be utilized for tasks such as the Attentional Blink (Lawson et al., 2002; Crewther et al., 2007) and Temporal Order Judgment (TOJ) tasks (Zackon et al., 1999) in which variations in the duration of stimulus presentation or duration interval that are pre-determined and allows full sampling of the psychometric function, or analysis of the shape of performance curve.

The use of non-motor psychophysical methods holds many advantages especially for use with stroke patients (List et al., 2008). Firstly, task implementation is simple, straightforward, effortless, and time efficient. More importantly, these tasks have the capacity to quantify the temporal efficiency of visual sensory and attentional processes that are not confounded by psychomotor response speed, the latter being orders of milliseconds greater than visual detection or discrimination. Therefore, for stroke patients where upper limb mobility and strength, and expressive verbal abilities are often compromised, these tasks may prove to be more reliable tools to screen and monitor the recovery of visually-driven deployment of attention over time (and in space)—a surrogate marker that would be expected to more accurately reflect the cognitive recovery of patients rather than physical recovery.

### Study aims

The focus of this systematic review was two-fold. Firstly, this review was aimed at investigating the degree to which temporal deployment of visual attention (i.e., the time course of attentional deployment) may be compromised post-stroke, and the neural markers associated with it. This aim would be addressed by exploring how performance on tasks were differentially affected by the following factors: (1) between patients with and without neglect; (2) between patients with lesions to different cerebral regions; and (3) between patients with lesions to different hemispheres, i.e., right-hemisphere damage (RHD) and left-hemisphere damage (LHD) patients.

In addition, this review aimed to identify studies that have employed *non-motor* psychophysical paradigms to characterize temporal deployment of attention following stroke. Identification of these studies was expected to provide knowledge of the extent to which non-motor contributions to performance are important in explaining temporal processing impairments. A list of common non-motor psychophysical paradigms and their associated methodologies is summarized in Table 1.

**Table 1:** **Summary of non-motor psychophysical tasks**.

**Task**	**Paradigm**	**Outcome of interest**
1.Attentional blink task 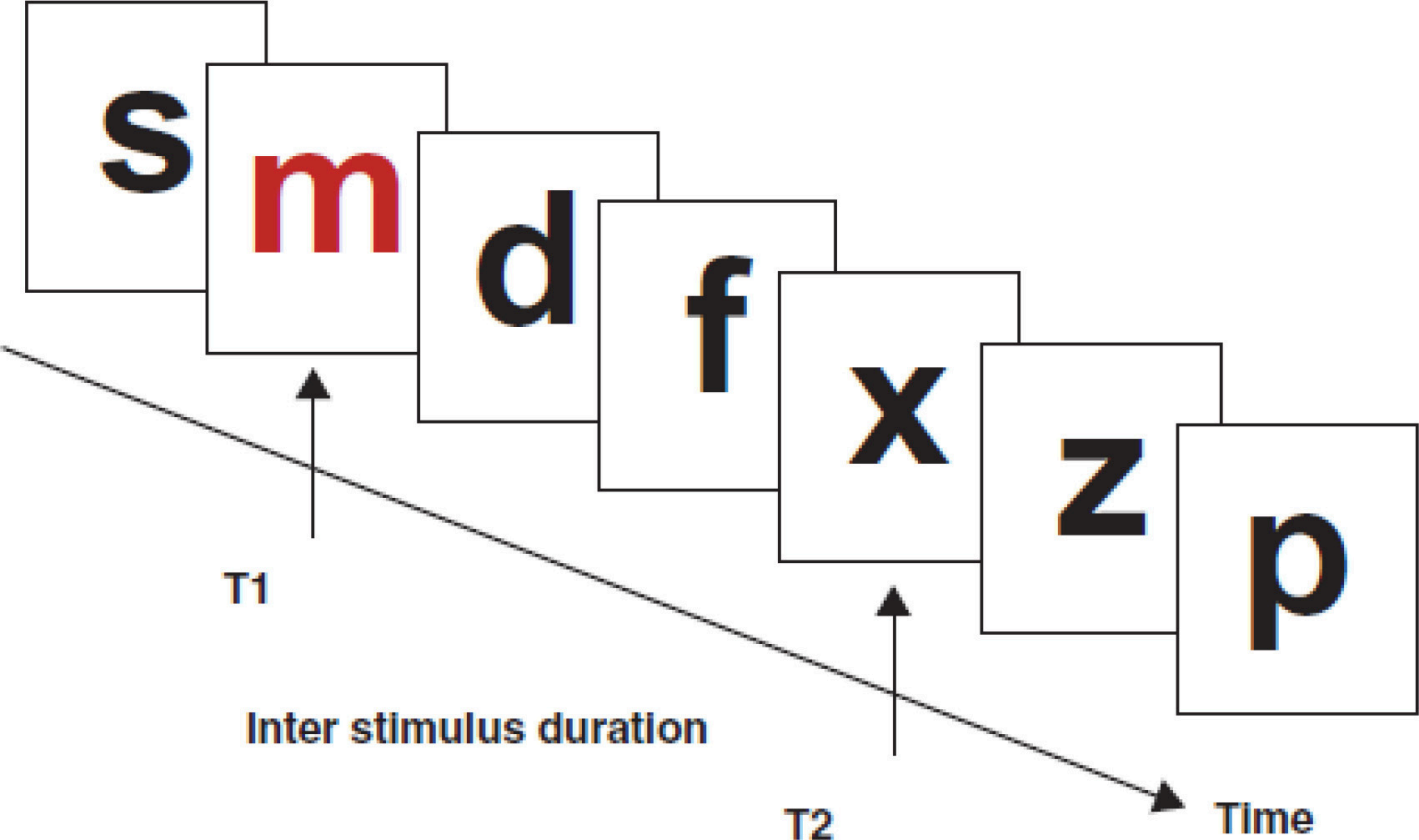 Attentional blink task involving presentation of a stream of stimuli. Adapted from Crewther et al. (2007).	Task involves presentation of a series of stimuli in the form of a rapid serial visual presentation (RSVP) paradigm. Two target items are embedded within the stream, with the second target item being presented at variable time intervals, or stimulus onset asynchronies (SOAs) across trials, following presentation of the first target item.	Attentional dwell time: the time interval required before one was able to accurately *determine the presence of the second target item*. “Attentional blink” refers to the phenomenon of missing the second target item, where the item was presented too soon after the first target item.
2. Inspection time task 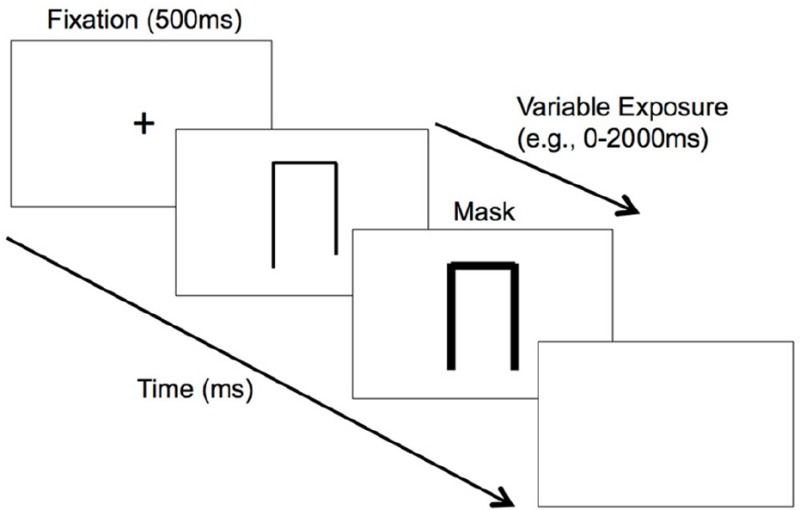 An inspection time task requiring detection of line length (i.e., which line is longer).	An adaptive threshold task that involves presentation of a stimulus across variable exposure durations. Exposure duration of stimulus adapts in accordance to the participant's response on the previous trial. If a correct response was given (i.e., correct detection of stimulus), stimulus exposure duration reduces on the next trial. Vice-versa for an incorrect response. Stimulus exposure duration is programmed to increase and decrease in step-sizes.	The threshold, or the minimal exposure duration required before one was able to accurately *detect the stimulus*. Task is programmed to measure the duration required to attend and encode stimulus into short term visual memory.
3. Change detection task (Visual paradigm) 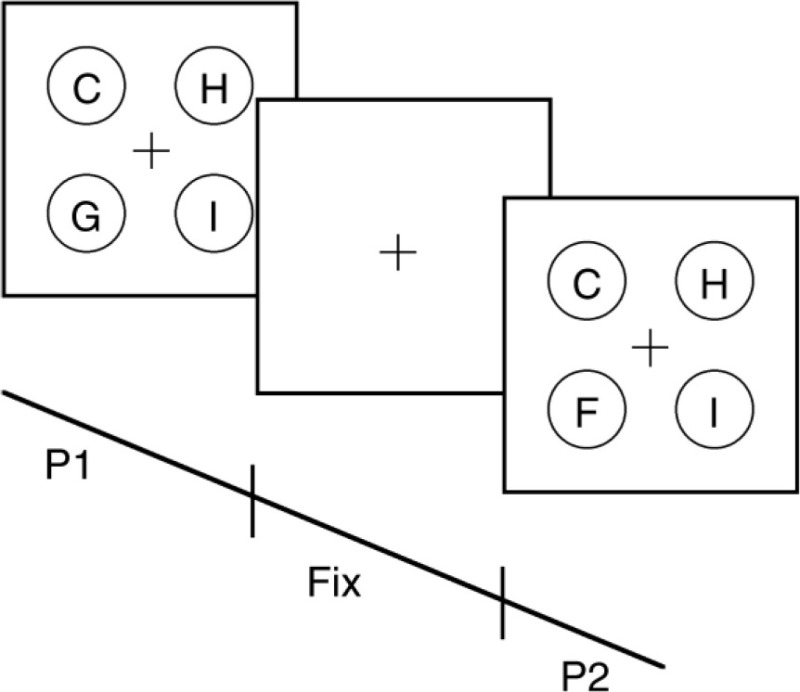 Illustration of a change detection task. Adapted from Rutkowski et al. (2003).	An adaptive threshold task that involves presentation of a set of stimuli across variable exposure durations, followed by presentation of a second set of stimuli following a fixed duration interval. Participants are required to determine whether stimuli has changed or remained the same as before. Exposure duration of the first stimuli set adapts in accordance to the participant's response on the previous trial. If a correct response was given (i.e., correct stimulus change detection), exposure duration reduces on the next trial. Vice-versa for an incorrect response. Exposure duration is programmed to increase and decrease in step-sizes.	The threshold, or minimal exposure duration required before one was able to accurately *detect a stimulus change*. Task is programmed to measure the duration required to attend and encode stimulus information into short-term visual memory, and to subsequently compare with the next stimuli set.
4. Temporal order judgment task (Visual paradigm) 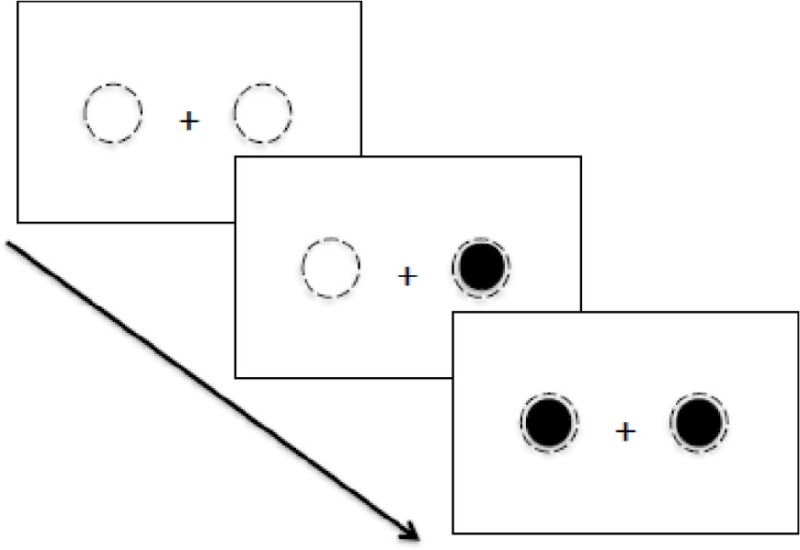 Temporal order judgment task requiring identification of the order of stimulus presentation.	Task involves presentation of two stimuli appearing, one to the left, and another to the right of fixation, either simultaneously, or at variable time intervals across trials. Participants are required to accurately determine the order in which the stimuli were presented.	The time interval required before one was able to accurately *determine the order* of stimuli presentation.

*Given the versatility of computerized measures, a variety of tasks exist in the literature, in which modifications may have been made to the underlying features and programming of the tasks, including but not limited to the form and location of stimulus presentation*.

Given that a variety of task paradigms and methodologies are utilized across studies, there is likely to be differences in levels of complexity and cognitive loading (e.g., where greater cognitive resources are required, tasks may extend beyond fundamental temporal processing of the visual stimulus), in stimulus form (e.g., shapes, color, symbolic information etc.) and in spatial location of stimulus presentation (e.g., centrally or within each visual hemi-space). As a result of this variability, coupled with the heterogeneous nature of stroke sample characteristics, a qualitative review of studies from this systematic search was deemed to be more methodologically feasible than a meta-analytic approach in addressing the current aims.

## Methods

The 2009 PRISMA (Preferred Reporting Items for Systematic Reviews and Meta Analyses) checklist was used to guide the reporting of this systematic review (Moher et al., 2009).

### Literature search strategy

A literature search was performed on PsycINFO and Medline databases, comprising studies up until the 11th September 2015. The search strategy was developed in collaboration with a research librarian from La Trobe University. Keywords and search terms associated with the paradigms of interest were identified, including “attention^*^ blink” OR “change detection” OR “change blindness” OR (“reaction time” AND attention”) OR “inspection time” OR “visual detection” OR “temporal attention” OR “temporal order judgment.” Search terms that were broad in meaning (e.g., “visual attention” and “visual processing”) were not included, as preliminary search trials using these terms led to a considerably wide range of unrelated studies. Next, keywords and search terms that relate to the population of interest were identified—this includes “stroke” OR “lacunar stroke” OR “cerebral ischemia” OR “brain ischemia” OR “cerebrovascular accident” OR “neglect” OR “transient ischemic attack.” The resulting collection of studies from each theme were then combined (i.e., AND).

The above search led to a total of 534 articles. Of these, articles were excluded if they: (1) were review studies, (2) involved only animal research, and/or (3) were focused on rehabilitation aspects. Apart from using keywords and search terms to eliminate these studies (i.e., “review” OR “literature review” OR “training” OR “computer training” OR “computer user training” OR “rehabilitation” OR “cognitive rehabilitation” OR “animals”), they were concurrently reviewed by their title and abstract to confirm exclusion. Resulting articles were further limited to English language and peer-reviewed journals, with duplicate articles removed, resulting in 334 articles.

**Figure 1:**
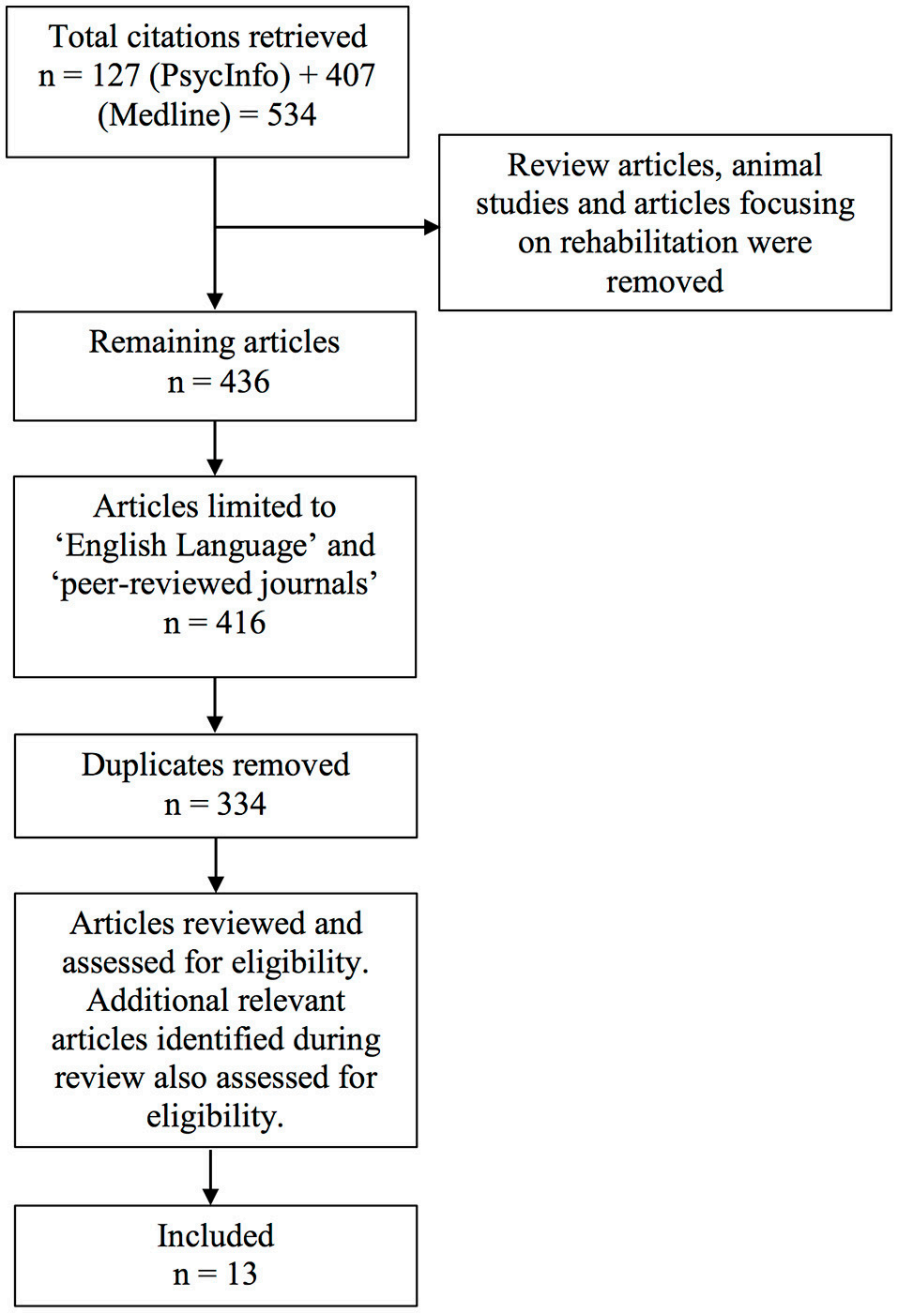
**Flow diagram of review process**.

Finally, the abstracts and methodological content of the above collection of articles were individually reviewed by two researchers (EL and RL) to identify those that met the selection criteria (as per Section Selection Criteria). Where additional studies were identified during the review process, they were similarly examined to determine eligibility for inclusion. A flow diagram of the search process is presented in Figure 1.

### Selection criteria

To address the aims of the current review, studies had to fulfill several inclusion criteria:

1. There should be a focus on the temporal deployment of attention in space, through the visual modality.
2. A computerized task should have been used, in which temporal deployment was measured by varying the stimulus exposure duration, stimulus onset asynchronies, or the time elapsed (i.e., duration interval) between presentation of two consecutive stimuli or sets of stimuli. Note that this differs from situations where stimulus exposure time was manipulated primarily for calibration purposes, and where the final exposure duration was kept constant in experimental trials to avoid ceiling and floor effects (Bonato et al., 2010).
3. The task employed should be a non-motor task, whereby a psychomotor response component (e.g., saccadic, verbal, or motor reaction time) was not required.
4. Studies should have recruited cerebral ischemic stroke and/or TIA patients.

Criterion (4) was applied to the selection criteria, given that the use of the term “stroke” also led to studies where “stroke” was interpreted in a non-neurological context (e.g., number of strokes in Chinese calligraphy, number of strokes in a squash game, etc.). In addition, the use of criterion (4) enabled the exclusion of studies that had made reference to, but did not recruit stroke patients (i.e., focus was on a different population). Finally, articles were excluded if the aim of the articles was to investigate the effects of an independent variable on attention (e.g., the effects of emotional stimuli/nicotine/phasic alerting/prism adaptation on attention/TOJ, etc.). This exclusion criterion was applied to identify only studies that investigated residual deployment abilities as a result of a brain injury rather than an experimental manipulation.

### Data extraction and synthesis

Information relevant to the current review was extracted, including participant groups and characteristics (i.e., sample size and age), mean time post-stroke, tasks employed, study aims, the dependent variable or the variable of interest, and the main findings. Studies were grouped into thematic categories based on their primary aim, and more importantly, based on the factors that were taken into consideration by the authors when recruiting and categorizing patients into stroke subgroups. For example, studies that investigated the role of neglect on task performance mostly categorized patients by neglect and non-neglect subgroups, while those that investigated the role of lesion area on task performance, categorized patients into subgroups based on the area of the ischemic lesion.

## Results

### Search results

Application of the above selection criteria led to identification of a total of 13 studies involving stroke patients. While Transient Ischemic Attack (TIA) had been included as a search term to determine the presence of studies utilizing TIA patients, no studies were retrieved that involved these patients. Of the 13 selected articles, one was identified from the reference list during the reviewing of the articles. Ten of the thirteen articles were experimental studies, while the remaining three were case studies. For the purpose of the current review, only results from experimental studies were evaluated collectively, since conclusions may not be reliably drawn upon from case studies due to the absence of a concurrent control group. Data pertaining to the experimental studies are presented in Table 2 and discussed in the subsequent sections, while data pertaining to case studies (Di Pellegrino et al., 1998; Hillstrom et al., 2004; Snyder and Chatterjee, 2004) are presented in Table 3.

**Table 2:** **Summary of identified experimental studies**.

**Authors**	**Participants (*n*, mean age, range/SD)**	**Mean time since injury (range/SD)**	**Study aims**	**Tasks**	**Variable(s) of interest**	**Findings**
Husain et al., 1997	RHD without neglect (*n* = 8, 64, −)	1 month (−).	To investigate temporal attention in patients with neglect	Attentional Blink (RSVP procedure)	Accuracy in detecting second target stimulus across variables SOAs	Neglect patients demonstrated an attentional blink that was three times longer than non-neglect patients. Performances of non-neglect patients were not significantly different to that of controls.
	RHD with neglect (*n* = 8, 64, −)					
	HC (*n* = 10, 73, −)					
Rizzo et al., 2001	Stroke patients: 9 RHD, 3 LHD, 1 bilateral (*n* = 13, 53.5, ±15.2)	Not indicated, but lesions reported to be chronic.	To investigate whether attentional blink occurs outside the neglect syndrome, and persists into the chronic phase of injury	Attentional Blink (RSVP procedure)	Attentional blink length and magnitude	Increased attentional blink (length and magnitude) occurred with unilateral lesions of either hemisphere, and also in the absence of neglect. Effects persisted into the chronic phase of injury, lasting years after the initial lesion.
	HC (*n* = 9, 65.2, ±2.2)					
	Three of the stroke patients had neglect					
Shapiro et al., 2002	SPL lesion: all RHD (*n* = 4, −, −)	No longer than 6 weeks post-stroke.	To investigate visuo-temporal attention in patients with/without neglect.	Attentional Blink (RSVP procedure)	Accuracy in detecting second target stimulus across variable SOAs.	Irrespective of whether neglect was present/absent, damage to the IPL and STG resulted in a protracted attentional blink. However, IPL and STG patients with left-side neglect (i.e., RHD) performed worse than IPL and STG patients without neglect (i.e., LHD). Performance of SPL group was comparable to controls. IPL and STG play a role in non-spatial attention.
	IPL and STG lesion: 4 LHD and 3 RHD (*n* = 7, −, −)					
	HC (*n* = 10)^a^					
	Three of the IPL and STG patients had neglect.					
Sinnett et al., 2007	RHD with and without neglect (*n* = 7, 55, 36–74)	35 months (3–96).	To examine spatial attention across visual and auditory modalities^b^	Temporal Order Judgment (visual and auditory versions).	Just Noticeable Difference (JND): average SOA at which 75% accuracy across trials was achieved.	Minimum time required to achieve an accuracy of 75% across trials was significantly longer in RHD patients compared to controls, for presentations of visual stimuli. There was no significant difference in JND scores between patient and control groups, for presentations of auditory stimuli.
	HC (*n* = 18, 51, 38–80)					
List et al., 2008	Unilateral lesion patients: 13 RHD, 10 LHD (*n* = 23, 66, 37–88)	5.6 months (3–9).	To empirically validate a computerized procedure for implementation in the assessment of neglect.	Feature Search, Scattered Feature Search and Conjunction Search Task (adaptive staircase procedure).	Stimulus exposure duration required to achieve 75% accuracy. Threshold presentation time was calculated by averaging last eight reversals.	Controls demonstrated spatially symmetric performance. Patients demonstrated lateralized impairments that were greater in conjunction search compared to feature search. Lateralized deficits were greater in RHD patients compared to LHD patients in the conjunction search condition.
	HC (*n* = 12, 63, 52–70)					
	All patients had neglect.					
Godefroy et al., 2010	Stroke patients (*n* = 36, 54, ±13.8)	11 months (–).	To investigate the determinants of stroke-related action slowing.	Visual Inspection Time, amongst other tasks (finger tapping, simple and choice RT).	Minimum stimulus exposure duration required to achieve 80% accuracy.	Patients were slower on all tests except for choice RT. The main determinant of action slowing was lesion location. Visual inspection time correlated with right inferior parietal lobule lesions. Finger tapping correlated with left frontal middle gyrus and lenticular nucleus lesions. Simple RT correlated with right lenticular nucleus and posterior fossa lesions.
	HC (*n* = 36, 55, ±14.2)					
	There were no neglect patients.					
Arend et al., 2011	RHD with visual extinction (*n* = 5, 68, ±11.5)	Patients were recruited between 2 and 5 years post-stroke.	To investigate the neural basis of temporal binding.	RSVP of five stimulus presented either ipsi- or contralesionally.	Proportion of binding errors (when the letter before or after the target was reported as the target letter) for ipsi- and contralesional field.	Patients demonstrated significantly more binding errors compared to controls. Patients made significantly more binding errors for contralesional than ipsilesional stimuli.
	HC (*n* = 8, 67, ±6.7).					
Correani and Humphreys, 2011	Posterior parietal lesion patients^c^(*n* = 11, 66, −)	10.5 years (4–20).	To investigate attentional dwell time in patients with posterior parietal or frontal lesions.	Attentional Blink.	Accuracy in detecting and reporting features of second target stimulus across variable SOAs.	Both patient groups demonstrated significantly longer attentional dwell time compared to controls. Dwell time did not differ between patient groups.
	Frontal lesion patients^c^(*n* = 8, 60, −)					
	HC (*n* = 10, −, −)					
	There were no neglect patients.					
Roberts et al., 2012	Unilateral and bilateral stroke patients^d^(*n* = 24, 63.6, 34–76)	5.3 years (0.5–18).	To investigate spatial and temporal attention deficits in patients with left-side and right-side neglect, and in non-neglect patients.	Temporal Order Judgment.	Spatial score: measure of spatial bias.	Patients with left-side neglect demonstrated left spatial deficit along with poor temporal resolution (except for two patients with bilateral lesions). Patients with right-side neglect demonstrated normal spatial and temporal performance (except one patient with LHD extending into the white matter of the parietal lobe. Of those without neglect, only one patient demonstrated spatial and temporal deficits.
					Temporal score: temporal interval between the stimuli required to respond accurately.	
		HC (*n* = 8, 68, (60–80)				
		11 of the stroke patients had left-side neglect and 7 had right-side neglect.				
Russell et al., 2013^e^	RHD (*n* = 5, 66, 55–75)	3 months (−).	To assess spatial and temporal deficits in RHD patients without neglect, under conditions of low and high attention load.	Modified Attentional Blink, with second target presented either ipsi- or contralesionally.	Accuracy in detecting second target stimulus across variable SOAs.	Under conditions of high attention load, patients demonstrated an extended attention blink, which was worse for letters presented on the contralesional side.
	HC (*n* = 5, 65, 56–70)					
	There were no neglect patients.					

*SD, standard deviation; RHD, patients with right-hemisphere damage; LHD, patients with left-hemisphere damage; HC, healthy controls; RSVP, rapid serial visual presentation; SPL, superior parietal lobule; IPL, inferior parietal lobule; STG, superior temporal gyrus; JND, just noticeable difference; RT, reaction time; SOA, stimulus onset asynchrony. Dashes indicate where descriptive statistics were not reported in article*.

a *Control participants reported in this study were the same participants reported in the study by Husain et al. (1997)*.

b *While aim of study was to examine spatial attention, temporal characteristics of performance was also reported*.

c *Five of the 19 patients did not suffer a stroke. One had an aneurysm, one had encephalitis, two suffered from carbon monoxide poisoning, and one was diagnosed with posterior atrophy*.

d *Three of the 24 patients did not suffer a stroke. Two had encephalitis, while one other suffered from anoxia*.

e *Only Study 1 was reported. Study 2 investigated attention in healthy aging*.

**Table 3:** **Summary of identified case studies**.

**Authors**	**Patient case**	**Study aims**	**Tasks**	**Variable(s) of interest**	**Findings**
Di Pellegrino et al., 1998	Patient FB (65 years, male, right MCA infarct, seen 5 months post-stroke, neglect resolved at time of assessment while visual extinction still present).	To investigate attentional dwell time in a patient with unilateral extinction.	Modified attentional blink, with both targets presented either ipsi- or contralesionally.	Accuracy in reporting second target across variable SOAs.	Duration required for identification of second target was twice longer, when targets were presented in the contralesional, compared to the ipsilesional field.
Snyder and Chatterjee, 2004	Patient AF (41 years, male, acute right temporal-parietal stroke, visual extinction with mild neglect present at time of assessment).	To investigate if temporal judgment of stimulus would be worse in the contra-, compared to ipsilesional space).	Temporal Order Judgment.	Percentage accuracy across variable SOAs.	Judgment of temporal order of successive ipsilesional stimuli was more accurate than for contralesional stimuli, with a longer refractory period required for presentation of contralesional stimuli.
Hillstrom et al., 2004	Patient SR (68 years, male, right MCA infarct involving inferior parietal, inferior frontal and temporal lobes, seen 7 months post-stroke, neglect present at time of assessment).	To investigate the spatio-temporal dynamics of directing attention, in the presence of neglect.	Modified attentional blink, with second target presented either ipsi- or contralesionally.	Accuracy in reporting second target across variable SOAs.	Patient required more time to identify second target when target was presented contralesionally (i.e., to the left), compared to ipsilesionally.

*SOA, stimulus onset asynchrony; MCA, middle cerebral artery*.

The primary reason for the exclusion of the majority of articles was that, although stroke patients were mentioned, they were not the clinical cohorts of interest, and were therefore not recruited into the studies. Secondly, a significant proportion of studies had a focus on other (non-visual) sensory modalities, including auditory attention and tactile attention. The current search also retrieved many studies that employed Posner tasks that had to be excluded, as this paradigm required psychomotor reaction time responses. Retrieval of the Posner studies was due to the use of the term “reaction time” during the search process. However, it was the authors' intention to include this term to maximize results, since the term has been used quite broadly and interchangeably in the literature.

### Characteristics of studies

A total of 159 stroke patients and 126 control participants were identified across the 10 experimental studies.

In regards to the aim of the studies and the way in which patients were recruited, three of the 10 studies were primarily aimed at comparing task performance between stroke patients with neglect and those without neglect (*n* = 29 with neglect, *n* = 24 without neglect, and *n* = 27 controls; Husain et al., 1997; Rizzo et al., 2001; Roberts et al., 2012), two studies investigated performances based on lesion location (*n* = 8 with frontal lesions, *n* = 22 with parietal lesions, and *n* = 20 controls; Shapiro et al., 2002; Correani and Humphreys, 2011), and four studies investigated performances by recruiting either RHD and/or LHD patients (*n* = 30 RHD, *n* = 10 LHD, and *n* = 43 controls; Sinnett et al., 2007; List et al., 2008; Arend et al., 2011; Russell et al., 2013). One remaining study investigated the performance of a heterogeneous stroke group relative to a control group, without categorizing patients into more specific subgroups (*n* = 36 patients, and *n* = 36 controls; Godefroy et al., 2010).

Note that, while a primary aim was present, factors such as neglect, lesion area, and side of lesion, were often considered concurrently in the recruitment process. Therefore, while Shapiro et al. (2002) investigated performances between patients with lesions to different brain regions, the authors also accounted for presence of neglect symptoms by recruiting patients with and without neglect. On the other hand, while the study by Correani and Humphreys (2011) was aimed at investigating temporal deployment differences based on lesion area, the authors recruited only non-neglect patients to ensure homogeneity of sample. In this context, we would consider the study by Correani and Humphreys (2011) to be equally valuable in informing the temporal dynamics of visual attentional orienting in non-neglect patients, as it would be for lesion area.

In regards to choice of paradigms, six studies employed a rapid serial visual presentation (RSVP) procedure to investigate the attentional blink phenomenon (Husain et al., 1997; Rizzo et al., 2001; Shapiro et al., 2002; Arend et al., 2011; Correani and Humphreys, 2011; Russell et al., 2013), two studies employed a TOJ task (Sinnett et al., 2007; Roberts et al., 2012), one employed a visual inspection task (Godefroy et al., 2010), and one other study employed a visual feature and conjunction search task via an adaptive staircase paradigm (List et al., 2008). Results are discussed in the subsequent sections based on thematic categories of neglect, lesion area, and lesion side. See Table 1 for a detailed explanation of relevant task paradigms.

### Task performance factored by neglect

Husain et al. (1997) employed the attentional blink task to quantify attentional blink length on 16 patients with RHD, 8 of whom displayed neglect (based on clinical presentation and performance on a shape-cancelation task) following a stroke to frontal, parietal and basal ganglia regions. With regards to the task, patients were required to detect the second target letter following detection of an initial letter, from an RSVP stream of letters. Findings from this study revealed an attentional blink in the neglect group that was three times longer compared to patients without neglect, while no difference in performances were observed between non-neglect patients and controls.

In contrast to the findings by Husain et al. (1997), subsequent studies have predominantly demonstrated impaired attentional blink, even in patients who were not clinically diagnosed with neglect. For example, Rizzo et al. (2001) characterized attentional dwell time using the attentional blink task in unilesional (comprising either right or left side lesions) stroke patients with and without neglect. The authors found a significantly prolonged attentional blink in patients compared to controls, irrespective of the presence or absence of neglect. These findings were also in line with attentional blink studies by Correani and Humphreys (2011) (mix of frontal and parietal lesion patients, lesion side not reported) and Russell et al. (2013) (only RHD patients), in which their patients without neglect exhibited significantly prolonged attentional blink compared to control participants. These studies are discussed further in the subsequent sections.

A further study by Roberts et al. (2012), aimed at investigating both spatial and temporal allocation deficits on a TOJ task, employed an exploratory approach with a less conservative recruitment criterion. Specifically, a lesion laterality index (measuring the extent of laterality of the lesion) was used, and identified 5 of 24 recruited patients as having a purely LHD, three with purely RHD, and the remaining patients as having bilateral lesions. On the TOJ task, patients were asked to determine the order of two letters appearing, one to the left and another to the right of fixation, at varying onset asynchronies between the two letters. In addition to spatial bias scores (that indicate neglect based on the TOJ task performance), temporal resolutions were compared between those with a spatial deficit (i.e., either right or left side neglect; *n* = 18), and those without (*n* = 6). Interestingly, results revealed differential temporal resolutions even amongst patients with neglect, whereby temporal deficits occurred alongside a left spatial deficit (presumably more right-lateralized lesions), but not in patients with a right spatial deficit. More interestingly, although temporal deficits were not observed in the group without neglect, one patient did demonstrate impaired temporal resolution, requiring a substantially longer time interval between stimuli to perform the task accurately. This latter finding provides further support for an abnormality in the temporal dynamics of attentional deployment, even in the absence of neglect.

### Task performance factored by lesion area

Correani and Humphreys (2011) conducted a study to specifically investigate whether the duration of the attentional blink was differentially affected by lesion site. Eleven non-neglect patients with posterior parietal lesions (lesions were reported to be located within the inferior parietal and superior temporal regions) and 8 patients with prefrontal lesions (lesions were reported to largely include the middle frontal gyrus) were therefore recruited, and performed a similar attentional blink task to that used by Husain et al. (1997) (as reported in Section Task Performance Factored by Neglect). Findings from this study revealed prolonged attentional blink in both patient groups compared to controls. Additionally, there was also no difference found in blink length between the two patient groups. On this basis, the temporal deficits were attributed to disruptions within the fronto-parietal network as a result of isolated lesions within inferior parietal areas (including the temporo-parietal junction) and the frontal cortex bilaterally (Correani and Humphreys, 2011).

In line with the above findings, Shapiro et al. (2002) examined the attentional blink in patients who, irrespective of the side of lesion, had a lesion to either the superior parietal lobule (SPL) (*n* = 4; all with RHD) or to both the inferior parietal lobule (IPL) and superior temporal gyrus (STG) (*n* = 7; four with LHD and three with RHD and neglect). Although, the performance of the SPL group was not significantly different to that of controls, attentional blink performance was found to be slower and significantly more impaired in the IPL and STG group. Importantly, the authors did not find any difference between neglect and non-neglect patients within the IPL and STG group, with patients exhibiting prolonged attentional blink irrespective of neglect. Based on these findings, the authors concluded that prolonged attentional blink was more likely to be driven by the location of the lesion, particularly regions comprising the IPL and STG (Shapiro et al., 2002). However, it remains unclear whether the IPL, or STG, *per se*, plays a more important role.

Clinical-anatomical correlations were performed in 2 of the 10 experimental studies identified in this review, and appeared to provide strong support for the aforementioned findings. The first study by Godefroy et al. (2010) involved administration of a visual inspection time task (in addition to several other non-psychophysical tasks) to investigate the mechanisms responsible for slowing of actions following stroke. This task involves central presentation of two vertical lines of different lengths. Patient participants were then required to identify the shorter of the two lines, with the exposure duration of the lines varying across trials, based on response accuracy of the previous trial. In this study, while all patients did not present with neglect symptoms, they were found to require a significantly longer stimulus exposure time compared to controls before they were able to achieve 80% accuracy in discriminating line length. This meant that they required a longer threshold time to efficiently deploy and allocate attention to the line stimuli following a stroke. More importantly, Godefroy et al. (2010) found a significant correlation between inspection time performance with only lesions of the right IPL (amongst 18 regions of interest), that is suggestive of a strong functional link between the right inferior parietal lobe with attentional deployment speed. A limitation with this study, however, is the lack of classification of the stroke cohort, including the side of lesion, and whether patients presented with unilateral or bilateral ischemic lesions.

The second clinical-anatomical correlation study by Roberts et al. (2012) (see previous section: Task Performance Factored by Neglect, for further details) revealed poor TOJ performance to be significantly correlated with damage to the right temporo-parietal lobe (comprising the post-central gyrus, angular gyrus, and superior temporal gyrus) and the cerebellum bilaterally. While the associations with cerebellar regions are likely to be a function of the cerebellum's role in sub-second timing (Ivry and Spencer, 2004; Koch et al., 2007; Aso et al., 2010), as argued by the authors, the associations with parietal and superior temporal regions again suggest a functional relationship between the speed of attentional deployment with these regions.

### Task performance factored by side of lesion

Temporal deficits of attention following a right-sided ischemic stroke have been demonstrated via use of several non-motor psychophysical tasks, as per studies that have been identified from the current systematic search. Sinnett et al. (2007), for example, revealed that RHD patients (some of which from this group had neglect symptoms) were poorer at judging the temporal order of two sequential stimulus presentations on a TOJ task compared to controls. This was particularly evidenced by a longer duration interval required between presentations of the two stimuli before patients were able to accurately determine the order of stimuli presentation. Similarly, in a study by Arend et al. (2011) that employed an RSVP task (involving presentation of a stream of five letters to the left or right of central fixation), the authors identified significantly greater temporal binding errors in five RHD patients compared to controls. This finding indicated generally poorer abilities by right-side lesion patients, in accurately judging the order of stimuli presentation. It should be noted in this study, that all five RHD patients also presented with visual extinction on confrontational testing, but not necessarily with unilateral spatial neglect (the latter was not examined).

A more recent study by Russell et al. (2013) considered the possibility of a laterality difference (between the two visual hemi-spaces) by employing a modified attentional blink paradigm, involving first, the presentation of a central target (T1), followed by a lateral (T2) target, either to the left or right of central fixation (and at either upper or lower quadrant positions). This design contrasted with previously mentioned RSVP studies, where all targets were presented at central fixation (Husain et al., 1997; Rizzo et al., 2001; Shapiro et al., 2002; Correani and Humphreys, 2011). Task demands were manipulated by the authors by additionally introducing a mask immediately following T1 (i.e., high attention load; Russell et al., 2013). Five RHD patients without neglect participated in this study, and notably, patients demonstrated significantly greater temporal deficits in identifying the second target especially when presented to the left, compared to the right of central visual space. Thus, despite the lesion being unilateral, a degree of compromise in the ability to efficiently deploy attention in space was present across both visual fields, although more pronounced in the contralesional visual field. This observed bilateral compromise to the temporal dynamics of attention following unilateral damage is not completely unexpected, given the implications of unilateral lesions on white matter and inter-hemispheric connectivity across callosal structures (Geschwind, 1965; Bartolomeo and Chokron, 2002).

A major limitation noted across studies thus far, was a tendency toward investigating only RHD patients. There was only one study by List et al. (2008) that recruited patients with both unilateral LHD (*n* = 10) and RHD (*n* = 13). However, a caveat pertaining to this study was that all patients were clinically diagnosed with neglect. List et al. (2008) employed an adaptive threshold visual search paradigm involving display of a set of stimuli, where participants were required to then search and identify a target on one side of the visual display. Exposure duration of the stimuli varied across trials via a staircase procedure, with two versions of the task being used, namely a feature search and a more attention-demanding conjunction search. Interestingly, lateralized patterns of temporal impairments (as reflected by a longer exposure time required for target identification) were demonstrated across all tasks for both RHD and LHD patients compared to controls. Furthermore, the authors found a greater extent of lateralized impairment in RHD compared to LHD patients on the more attentionally-demanding conjunction search paradigm. These findings were indicative of firstly, a greater degree of temporal deficit in the contralesional, compared to the ipsilesional visual field, and secondly, a degree of overall compromise that was greater following RHD, compared to LHD stroke.

## Discussion

The current systematic review was aimed at exploring the extent and nature of deficits related to visually-driven temporal deployment of attention in space post-stroke. In particular, this review sought to identify studies from the literature that employed non-motor psychophysical paradigms to characterize temporal deployment. A total of 13 studies were identified, 10 of which were experimental group studies and the remaining three were case studies.

An appraisal of task performance by thematic categories across these studies (i.e., presence of neglect, lesion location, and lesion side) revealed several noteworthy findings. Firstly, there appears to be strong evidence to suggest an important role of more inferior regions of the fronto-parietal network, including the IPL and STG, in facilitating efficient deployment of attention for subsequent stimulus identification. Correani and Humphreys (2011), in particular, revealed prolonged attentional blink in patients with frontal lesions only, as well as in patients with inferior parietal and superior temporal lesions. More importantly, when attentional blink was investigated between patients with specifically IPL lesions, and those with specifically SPL lesions, prolonged attentional blink was observed only in the IPL group (Shapiro et al., 2002).

Secondly, results from studies that investigated the role of neglect were broadly unanimous in demonstrating temporal deployment deficits, irrespective of whether neglect was present or otherwise. This was mostly observed in attentional blink studies, where a significantly longer time was required to efficiently deploy attention to facilitate target identification, even in patients without neglect (Rizzo et al., 2001; Shapiro et al., 2002; Correani and Humphreys, 2011). An anomaly, however, were the findings by Husain et al. (1997), whereby generally commensurate attentional blink performance between non-neglect patients and control subjects were demonstrated. This inconsistency may be due to confounding factors, with a likely explanation being that performance was more significantly driven or determined by the location of the lesion. In fact, lesion characterization was performed by the authors via computed tomography and revealed that four of the eight RHD patients without neglect suffered an infarct within the superior parietal lobe (two patients), temporal lobe (one), and the medial frontal lobe (one) while the remaining four had subcortical strokes (Husain et al., 1997). On the other hand, the eight RHD patients with neglect suffered infarcts to frontal and parietal regions, with the region of greatest overlap reportedly being the inferior parietal and inferior frontal cortices (Husain et al., 1997). Given strong evidence for the role of the inferior fronto-parietal regions in modulating the temporal dynamics of attention (Shapiro et al., 2002; Godefroy et al., 2010; Correani and Humphreys, 2011; Roberts et al., 2012), absence of lesions in these regions in non-neglect patients may thus explain the comparable performance between this patient group and controls.

A third point to note is that, amongst studies where laterality of the lesion was considered, compromises in task performance were consistently demonstrated following RHD (Sinnett et al., 2007; Arend et al., 2011; Russell et al., 2013). In addition, the results by Russell et al. (2013) further suggested an extent of temporal compromise across both visual hemifields, despite patients having only a unilateral right-side lesion. While this was the case, the degree of temporal compromise was documented to be greater in the contralesional compared to the ipsilesional visual space, as would be expected (List et al., 2008; Russell et al., 2013).

Finally, there is some evidence to indicate a greater extent of deployment deficit following RHD compared to LHD. For example, List et al. (2008) found neglect patients with unilateral RHD to display a greater degree of lateralized impairment compared to neglect patients with unilateral LHD. This laterality difference was similarly demonstrated by Roberts et al. (2012) on a TOJ task, where compromised task performance (reflecting prolonged deployment) were evident in patients presenting with a left spatial deficit, and not in those with a right spatial deficit (note however, that spatial deficit in this context was operationalized by the degree of lesion lateralization). Furthermore, clinical-anatomical correlations revealed significant associations between task performance with only right hemisphere lesions of the IPL and temporo-parietal regions (Godefroy et al., 2010; Roberts et al., 2012).

To an extent, findings gathered from this systematic review appear to build on existing postulations regarding the role of the right inferior parietal and frontal regions for non-spatial attention (Husain and Nachev, 2007; Corbetta and Shulman, 2011). In a review by Husain and Nachev (2007), the authors suggested that, while superior parietal regions play a predominant role in spatial attentional processing, more ventrally-located IPL and TPJ have a non-spatial role for sustaining attention, detecting salient targets within a stream of stimuli, and for controlling attention over time (Pardo et al., 1991; Linden et al., 1999; Adler et al., 2001). In the current review, we further identified the same regions to be implicated when individuals were engaged in tasks measuring the “timing” or “speed” of attention to a location in space. Therefore, while Husain and Nachev (2007) have considered the IPL and TPJ as non-spatial regions of attention, in our view, these regions are still closely associated with spatial attention, but they reflect the *temporal dynamics* of spatial attention more so.

The current findings are also consistent with current notions of a right-hemisphere dominant role for visuospatial attention (Coull and Nobre, 1998; Corbetta and Shulman, 2011; De Schotten et al., 2011; Bartolomeo et al., 2012). Previous functional imaging studies, particularly from the work of Corbetta and colleagues, have consistently revealed greater activity in the right inferior fronto-parietal regions compared to the left (Corbetta and Shulman, 2002, 2011)—these are regions responsible for exogenous, bottom-up attention to space. Similarly, novel diffusion weighted imaging techniques have shown the same, significant right lateralization of ventrally-located fronto-parietal white matter tracts (known to mediate spatial attention), particularly the SLF II and SLF III (De Schotten et al., 2011). In a recent study by the working group of Corbetta's, attention was further found to be better explained by a functional network account than by individual lesion locations, which parallels with the identification of white matter tracts that underpin attention (Siegel et al., 2016). Overall, the current findings not only provide support for a right-lateralization of visuospatial attention, but more specifically, a right-lateralization for the temporal dynamics of visuospatial attention.

While the emphasis of this review has mostly been on the temporal aspects of attentional orientation in space, it is essential to always consider this temporal aspect of spatial attention as a single construct, rather than as a construct composed of separate temporal and spatial domains. This is especially important when attempting to understand everyday functional deficits post-stroke, where the difficulties faced by neglect patients in representing their environment, for example, are due to poor spatiotemporal representations. The universality between the deployment of attention over a time course, and in space, may very well-explain why attentional deployment becomes gradually prolonged from non-neglected to neglected space (Di Pellegrino et al., 1998; Hillstrom et al., 2004), as well as why spatial working memory deficits are known to have a temporal component (i.e., due to the inability to keep track of spatial information over the course of time; Ferber and Danckert, 2006; Striemer et al., 2013). In fact, it could be the case that the temporal deployment of attention is an underlying factor that drives neglect behavior, such that when deployment speed becomes slow, it reaches a threshold where spatial symptoms of neglect begin to manifest.

In the face of the above findings, some limitations should be considered. Firstly, the range of tasks used across studies identified in this review differed, and as such, task complexity also varied. For example, some tasks required greater attentional demands (Russell et al., 2013), while some others involved presentation of lateral targets that required the ability to spatially shift attention (from one location to another) in addition to allocating attention over time (Roberts et al., 2012; Russell et al., 2013). Due to this complexity, caution should be taken in comparing results, especially between studies that utilized different paradigms. Another important gap in the literature that was identified from this review is the absence or almost non-existence of studies that have compared the performance of non-neglect RHD and LHD patients relative to controls. Although, List et al. (2008) examined the performance of RHD and LHD patients, only patients with neglect symptoms were recruited into the study. While other studies may have examined the performance of RHD patients without neglect (Sinnett et al., 2007; Russell et al., 2013), the absence of a LHD group meant that the performance of non-neglect LHD patients relative to non-neglect RHD patients could not be established.

In summary, the current findings indicate that deficits related to the temporal deployment of attention are associated with lesions to the inferior regions of the fronto-parietal network, with deficits occurring independently of neglect, and likely to be more exacerbated following insults to the right hemisphere. Notably, the regions shown to be responsible for this exogenously-driven temporal deployment appear to be consistent with regions of the Ventral Attention Network (Corbetta and Shulman, 2002; Fox et al., 2006) that are often affected in neglect. Yet, temporal deployment deficits may occur even in the *absence* of neglect, potentially suggesting its value as a likely cognitive marker or predictor of neglect—this is an area that will likely benefit from further exploration. Overall, the current study serves to reinforce the value of using non-motor psychophysical paradigms to better measure the temporal efficiency of attentional deployment in space. These findings have significant implications to clinicians and patients alike, as more targeted cognitive rehabilitation could be developed with early identification of such temporal deficits.

## Author contributions

EL designed the study, conducted the systematic search and review, and prepared the manuscript. RL assisted with article evaluation and filtering, and made important theoretical contributions. SC designed the study and made important theoretical contributions. All authors were involved in the proofreading and revision of this manuscript. All authors agree to be accountable for the content of the work.

### Conflict of interest statement

The authors declare that the research was conducted in the absence of any commercial or financial relationships that could be construed as a potential conflict of interest.
